# Habitat‐Forming Macroalgae Show Greater Resilience to Marine Heatwaves Than Models Predict, Yet Chronic Ocean Warming Remains Poised to Deliver Considerable Future Declines

**DOI:** 10.1002/ece3.72604

**Published:** 2025-12-11

**Authors:** T. R. Davis, C. Champion, M. A. Coleman

**Affiliations:** ^1^ Fisheries Research, Marine Ecosystems NSW Department of Primary Industries and Regional Development Coffs Harbour New South Wales Australia; ^2^ National Marine Science Centre Southern Cross University Coffs Harbour New South Wales Australia

**Keywords:** Australia, bull kelp, climate change, crayweed, *Durvillaea*, *Ecklonia radiata*, kelp, *Phyllospora comosa*, species distribution model

## Abstract

Temperate macroalgal forests globally have undergone climate‐induced range contractions and localised extinctions in response to warming and marine heatwaves, with further significant losses projected to occur. Managing these foundational habitats into the future requires improved understanding of the expected changes in distributions and the timeframes over which these changes are likely to occur. Here, we combine observations at species range edges with species distribution models to examine the impacts of marine heatwaves and scrutinise the potential impacts of future climate scenarios on macroalgal distributions for inshore waters along the temperate southern Australian coastline. We compare modelled to realised changes in distributions at the equatorward range edge of three major habitat‐forming macroalgal species in Australia, *Ecklonia radiata*, *Phyllospora comosa* and *Durvillaea* spp. Monitoring revealed that no substantial range contractions occurred at the equatorward range edge for these species between 2019 and 2024, despite several moderate to strong marine heatwaves during this period. However, species distribution model predictions for this period indicated that marine heatwaves (MHWs) induced considerable temporary reductions (e.g., 30%–70%) in environmental suitability for these species, implying greater resilience to MHWs than models suggest. Future projections indicate that long‐term ocean warming may cause moderate (8–152 km for *E. radiata* under RCP2.6) to severe (832 km for *Durvillaea* under RCP8.5) range contractions in eastern Australia by 2100. Projections may overestimate macroalgal losses, as they do not consider factors that confer resilience to species under short‐term extremes (e.g., local adaptation potentially delaying projected contractions at the warm range edge). Considering spatial variation in adaptation is therefore essential to refine models and generate more realistic projections of climate‐mediated macroalgal loss.

## Graphical abstract

Field observations and species distribution models were used to examine the impacts of marine heatwaves and future climate scenarios on kelp distributions along the temperate southern Australian coastline. Monitoring revealed that no substantial range contractions occurred between 2019 and 2024, while future projections indicate that ocean warming may cause range contractions in eastern Australia by 2100.

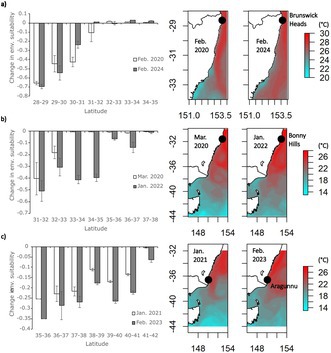

## 1 Introduction

Globally, temperate macroalgal forests are undergoing climate‐induced range shifts, contractions and localised extinctions, due to warming oceans and marine heatwaves (e.g., Filbee‐Dexter and Wernberg 2018; Filbee‐Dexter et al. 2020; Wernberg 2021; Straub et al. 2022). Generally, temperate macroalgal species are shifting poleward as the world's oceans warm, with equatorward edges of macroalgal forests transforming into ecosystems dominated by turfing algae and urchin barrens (Filbee‐Dexter and Scheibling 2014; Filbee‐Dexter and Wernberg 2018). However, in Australia macroalgal species have limited capacity to undergo poleward range shifts, due to a lack of suitable shallow reef habitat south of Australia's continental shelf. Rather, macroalgal forests face contractions and potentially localised extinctions at their equatorward (northern) range edges which are not offset by range extensions at the poleward range edge due to the lack of suitable reef habitat (Martínez et al. 2018; Davis, Champion, and Coleman 2022).

The macroalgal forests in southern Australia are the foundation of temperate marine ecosystems, producing food and shelter for a broad range of other species, including fishes and invertebrates (Bennett et al. 2016; Coleman and Wernberg 2017; Wernberg et al. 2019). Consequently, any range contractions of these forests will generate substantial negative economic, environmental, social and cultural consequences (Eger et al. 2023). Macroalgal forests dominate the Great Southern Reef (GSR), which extends > 8000 km along Australia's southern shoreline, where they contribute an estimated AU$6–10 billion per year to the Australian economy (Bennett et al. 2016; Eger et al. 2023). Worryingly, this region is a global warming hotspot (Hobday and Pecl 2014) and has already undergone several climate‐mediated declines in macroalgae (e.g., Wernberg et al. 2016; Vergés et al. 2016; Young et al. 2023). Understanding potential future range contractions for these key taxa is essential to guide management and conservation actions aimed at mitigating environmental and economic losses.

Substantial range contractions are projected for many species of macroalgae on the GSR in response to rising ocean temperatures (e.g., Martínez et al. 2018; Castro et al. 2020; Davis, Champion, and Coleman 2022). However, the estimated magnitudes of the range contractions vary substantially among studies, species, climate change scenarios and regions, creating uncertainty for marine managers. For example, Martínez et al. (2018) project a 71% reduction in the distribution of *Ecklonia radiata* in Australia by 2100, under representative concentration pathway (RCP) 6.0 (IPCC 2014), equating to a ~1100 km range contraction in eastern Australia. For the same species, Castro et al. (2020) estimate a range contraction of ~530 km by 2100 under the more severe RCP 8.5 scenario. Whereas Davis, Champion, and Coleman (2022) project an even lower range contraction of ~275 km by 2100 under RCP 8.5 by incorporating the impact of changing ecological interactions into models. Given this variability and uncertainty in future projections of species range contractions, it is important that models are ground‐truthed with empirical data, obtained by monitoring temporal distributional change, and utilise the latest climate projections. In particular, the upper thermal threshold for species survival may be higher than the maximum temperature at which they reside (the commonly used value in modelling) due to the occurrence of extreme events that lead to selection (Coleman and Wernberg 2020). For example, extreme marine heatwaves (MHWs; Hobday et al. 2018) are acute thermal events, that have precipitated substantial range contractions for *E. radiata* in Western Australia (Wernberg et al. 2016; Wernberg 2021) but have resulted in only partial mortality and genetic changes elsewhere in its Western range, suggesting that selection is occurring (Coleman, Minne, et al. 2020). Understanding how thermal events of differing magnitudes and durations impact macroalgal populations, based on empirical data, is vital for identifying realised thermal thresholds and parameterising models to accurately forecast change.

Here, we use 6 years of monitoring and environmental data to assess the impacts of acute thermal events (MHWs) on major habitat‐forming macroalgae species at their range edges in eastern Australia. We used this data to ground‐truth modelled predictions of distributional change. The species examined were *Ecklonia radiata*, the macroalga that dominates the GSR, *Phyllospora comosa*, which is an important habitat‐forming macroalga geographically restricted to south‐eastern Australia (Coleman and Wernberg 2017) and the closely related group *Durvillaea* spp. (*Durvillaea*), which was recently split into two species: *Durvillaea amatheiae* and *Durvillaea potatorum* (Weber et al. 2017) that are also restricted to south‐eastern Australia. These species were monitored at their equatorward range edge, from 2019 to 2024, with MHW effects quantified over this same period. Additionally, future projections of changes to the distributions of these species were made, for the period 2090–2100, using species distribution models (SDMs) developed for each species/group under RCP 2.6, 6.0 and 8.5 (IPCC 2014) future climate scenarios.

## 2 Methods

### 2.1 Modelling Extant Distribution

Macroalgal SDMs were developed for Australia using maximum entropy (Maxent) modelling, implemented using the ‘maxent’ package (Phillips et al. 2006) in R (R Core Team 2022). Maxent has been widely utilised to assess the effects of environmental change on species distributions (Merow et al. 2013) and was selected for use in this study as it (1) enabled SDMs to be developed using presence‐only data (Elith et al. 2011), (2) allows SDMs to be built using multiple non‐linear explanatory predictor variables (predictors), which are expected at the continental scale being examined and (3) has been applied in previous research examining range contractions in Australian macroalgae (Martínez et al. 2018) and therefore maximises comparability with existing research. The methods used in the current study were selected to conform with the current best practices for developing SDMs with Maxent (Merow et al. 2013; Radosavljevic and Anderson 2014). Species presence data used in Maxent modelling encompassed the temporal range 2000–2024 and were accessed from the Global Biodiversity Information Facility (GBIF 2023).

The extent of the study area for each macroalgal SDM was determined from the historical distribution of each species in Australia. Study areas in Maxent modelling should ideally cover the full environmental range of the species examined plus adjacent areas accessible via dispersal (Elith et al. 2011; Merow et al. 2013). Consequently, study areas encompassed the historical distribution of the target species, plus a 5.0° buffer, thereby capturing data across the full environmental gradient for all potential current and future presence locations. Additionally, study areas were restricted to the photic layer (i.e., to depths < 200 m), to exclude deeper offshore waters from models, with macroalgal occurrence limited to those depths where light is available for photosynthesis (Markager and Sand‐Jensen 1992). Note that model projections were not sensitive to the selected maximum model depth (i.e., 200 m) due to the non‐linear curves used to fit the relationships between species occurrence and depth.

**FIGURE 1:**
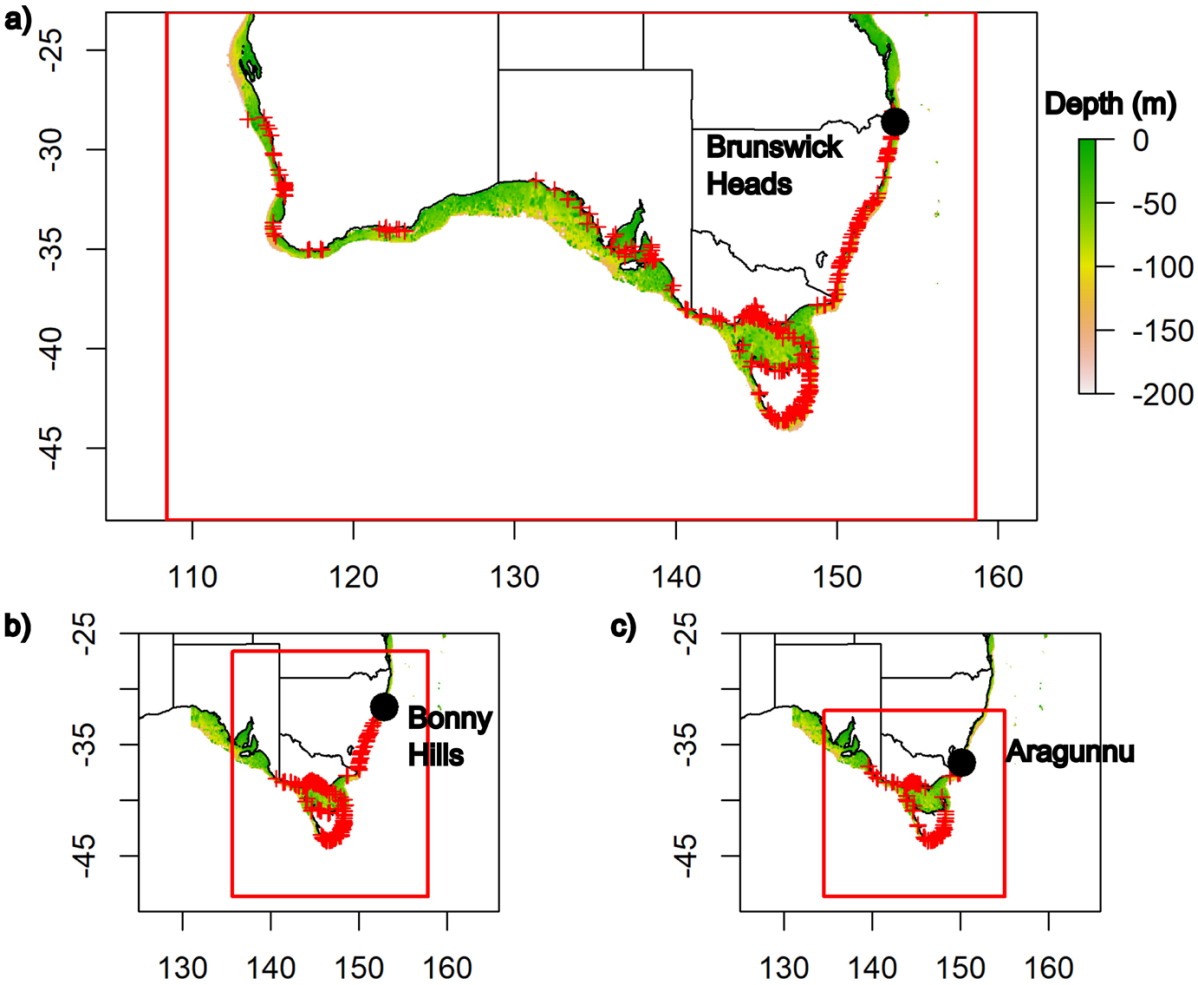
Study areas (red rectangles) for (a) *Ecklonia radiata*, (b) *Phyllospora comosa* and (c) *Durvillaea* spp. Red crosses indicate species presence records as obtained from GBIF. Contours indicate water depth (m). Black dots with site names represent the current equatorward limit of distribution.

For *E. radiata* , the current species distribution covers the entire southern coastline of Australia, with the species generally restricted to latitudes > 28.0° S. The study area for this species therefore extended from 108° to 159° E, 23.0° to 49.0° S and from shore to the 200 m depth contour (Figure 1a). For *P. comosa* , the current species distribution covers the south‐eastern corner of mainland Australia and Tasmania, with the species generally restricted to latitudes > 31.5° S and longitudes > 140° E. The study area for this species therefore extended from 135° to 158° E, 26.5° to 49.0° S and from shore to the 200 m depth contour (Figure 1b). Finally, for *Durvillaea*, the current species distribution is restricted to the south‐eastern corner of mainland Australia and Tasmania, with the species generally restricted to latitudes > 36.5° S and longitudes > 140° E. The study area for this species therefore extended from 135° to 155° E, 31.5° to 49.0° S and from shore to the 200 m depth contour (Figure 1c). We note that the species described here as *Durvillaea* spp. was historically called *Durvillaea potatorum* but was recently split into two separate species *Durvillaea potatorum* and *Durvillaea amatheiae* (Weber et al. 2017). These two species have been treated as a single species complex (i.e., *Durvillaea* spp.) for the purposes of the current study, due to the close genetic links between these two species, their overlapping ranges and their lack of distinction in historical presence records (Weber et al. 2017).

### 2.2 Model Development

Macroalgal presence data from GBIF were screened to exclude (1) records with no location coordinate information, (2) records where the location was on land and (3) records outside of Australia. Furthermore, records collected prior to 2000 were excluded to be consistent with the period over which historical environmental data were available. These screening steps resulted in 33,549 presence records for *E. radiata* , 17,628 for *P. comosa* and 723 for *Durvillaea*. To compensate for a priori assumed spatial autocorrelations in presence data, multiple records within each 0.05° cell (~5 km) were combined to give a single record, as recommended for filtering biological data (Johnston et al. 2019). The combination or removal of presence records within close proximity has been shown to be an appropriate method for handling spatial autocorrelation structures within species occurrence datasets (Brodie et al. 2015; Champion et al. 2021). Pooling data resulted in a reduced dataset for model development consisting of 326 presence records for *E. radiata* , 182 for *P. comosa* and 80 for *Durvillaea* (Figure 1). From these presence records, 70% were used for model training and 30% were retained as a cross‐validation dataset for model testing as per Martínez et al. (2018).

### 2.3 Environmental Predictor Variables

It is important that predictor variables used in SDM development have direct links to the physiological and ecological requirements of the species being examined (Elith et al. 2011; Merow et al. 2013). Thus, eight explanatory variables were a priori selected for initial inclusion in SDMs, based on their established links to macroalgal distributions, with data for these variables extracted for the period 2000–2020 from Bio‐ORACLE (Tyberghein et al. 2012; Assis et al. 2024). These were: (1) long‐term average maximum monthly SST (MaxTemp, °C); (2) long‐term average minimum monthly SST (MinTemp, °C); (3) annual average photosynthetically available radiation (PAR, E.m^−2^.day^−1^); (4) annual average nitrate availability (nitrate, mmol.m^−3^); (5) annual average phosphate availability (phosphate, mmol.m^−3^); (6) minimum water depth (depth, m); (7) Annual average salinity (salinity); and (8) mean annual current speed (current, m.s^−1^).

The following justifications formed the basis for the a priori selection of these explanatory variables. MaxTemp and MinTemp were selected as changes in temperature can cause large changes to physiological processes and the persistence of macroalgae (Mabin et al. 2013). Depth is a proxy for a range of depth‐varying environmental conditions of importance to macroalgae (e.g., irradiance, temperature) and has frequently been used in macroalgae SDMs (e.g., Young et al. 2015, Williams et al. 2020). Nitrate, phosphate, salinity and PAR all influence macroalgae growth and physiology (Fredersdorf et al. 2009; Mabin et al. 2013; Davis, Larkin, et al. 2022), while current strength plays an important role in nutrient supply and macroalgal sporophyte dispersal (Coleman et al. 2011).

**FIGURE 2:**
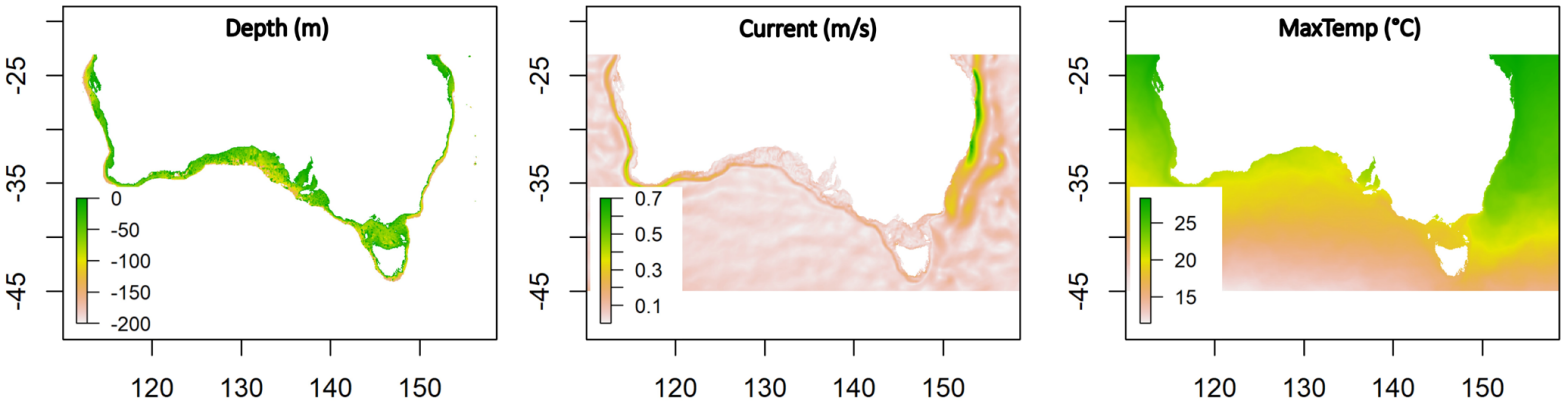
Maps of explanatory variables over the 2000–2020 period retained for Maxent modelling of macroalgal species distributions.

Prior to model fitting, all explanatory variables were assessed for collinearity, as is recommended for the development of Maxent models (Merow et al. 2013), with variables with Pearson correlation coefficients (|*r*|) > 0.7 combined, as recommended by Dormann et al. (2013). Temperature variables were found to be highly correlated and were also highly correlated with nitrate, phosphate, PAR and salinity (|*r*| > 0.81, all correlations). Consequently, MaxTemp was retained in models, acting as a proxy for this entire group of highly correlated variables, with MaxTemp selected to represent this group due to the known importance of upper temperatures in setting macroalgal distribution limits in Australia (Wernberg 2021). Consequently, after collinearity assessments three variables were retained for model development (Figure 2); long‐term average maximum monthly SST (MaxTemp, °C), minimum water depth (Depth, m) and mean annual current speed (Current, m.s^−1^).

### 2.4 Model Selection

Simpler models often provide similar performance to more complex models if they are implemented using a priori selected predictor variables, especially when feature selection and increased regularisation are tested (Merow et al. 2013), with simpler models preferred for modelling as they are less prone to overfitting the data. Consequently, to ensure the simplest and best performing models were selected for use in species redistribution assessments, a series of model trials were conducted. Firstly, the variables used in each SDM were explored through backward stepwise selection, which involved successively removing the individual variables that explained the least amount of variance. The area under the curve (AUC) of a receiver operating characteristic (ROC) plot was used to inform model variable selection among the resulting options, where the model associated with the highest AUC when tested against the cross‐validation dataset for each target species was selected as the optimal SDM. Secondly, the features used to construct each SDM in Maxent were explored by evaluating two model types: (1) using quadratic features (Quadratic model) as recommended by Merow et al. (2013); and (2) using the Maxent default combination of linear, quadratic, product and hinge features (LQPH model). Thirdly, different regularisation coefficient values were tested, as recommended by Merow et al. (2013), with coefficients of 1.0 (default) and 2.0 trialled. Regularisation coefficients provide a means of restricting model curve complexity, with smaller values allowing greater complexity by imposing fewer restrictions. The best performing model configuration (i.e., highest Test AUC value) for all species used the explanatory variables Depth, Current and MaxTemp with default LQPH features and the default regularisation coefficient of 1.0.

### 2.5 Climate Change Projection Data Source and Application

Long‐term projections of species' future distributions were developed for the period 2090–2100 under RCP 2.6, 6.0 and 8.5 scenarios (IPCC 2014), with the latter most closely aligning with current climate trends (Schwalm et al. 2020). Future data for MaxTemp and current speed variables were extracted from the Bio‐ORACLE online database (Tyberghein et al. 2012; Assis et al. 2024). Based on these data, coastal water temperatures in eastern Australia are projected to increase by an average of 0.8°C, 2.3°C and 3.4°C under RCPs 2.6, 6.0 and 8.5, respectively. Future changes to nitrates, phosphates, salinity and PAR were assumed to be adequately represented by changes to MaxTemp. This modelling decision is a significant simplification, based on the assumption that present‐day correlations among environmental variables will be retained under future climate change scenarios.

### 2.6 Presence Threshold

To estimate future changes to species distributions (particularly at range edges), it was necessary to convert probabilities of species occurrence, as calculated by Maxent, into binary species presence/absence data. Conversion of probability predictions to estimates of species presence and absence is achieved through the application of thresholds, with probabilities above the threshold taken to imply species presence and values below the threshold taken to imply species absence (Lawson et al. 2014). Here two different approaches were used to define the presence threshold, to allow assessment of the sensitivity of projected future species distributions to this value.

The first presence threshold applied was defined using Equal Training Sensitivity and Specificity criteria (ETSS, Table 1), calculated by Maxent, as applied by Martínez et al. (2018). Sensitivity provides a measure of how well the SDM identifies true presences, while specificity provides a measure of how well the SDM identifies true absences, with the ETSS threshold aiming to maximise projections of true presences and true absences.

**TABLE 1:** Equal training sensitivity and specificity (ETSS) and range limit (RL) thresholds used to define species presence for the best Maxent models developed for macroalgal species in Australia.

Model	ETSS threshold	RL threshold
*Ecklonia radiata*	0.190	0.407
*Phyllospora comosa*	0.214	0.594
*Durvillaea* spp.	0.224	0.162

The second presence threshold applied was determined using a Range Limit criteria (RL, Table 1). For this, the threshold was set at the maximum probability of occurrence in cells north of the current equatorward range limit for the species in eastern Australia (Brunswick Heads; functional range limit [28.6° S] for *E. radiata* , Bonny Hills [31.6° S] for *P. comosa* and Aragunnu [36.6° S] for *Durvillaea*). This approach ensured that each species was not predicted to be present in locations north of its current equatorward distributional limit, thereby ensuring that the predicted current distribution was consistent with the realised current distribution and thus maximising the ecological realism of future projections.

### 2.7 Assessment of Realised Marine Heatwave Impacts

The potential impacts of MHWs were assessed for each species using data on MHWs that have occurred since 2019 (matching our monitoring data) at the equatorward range edge for each species (Table 2). MHW data were extracted from the MHW tracker portal (www.marineheatwaves.org, accessed 20/01/2025) for MHWs with prolonged durations (i.e., > 10 days) which occurred over summer, with the baseline long‐term climatological mean calculated from 1982 to 2011. Data for summer MHWs were used as water temperatures are highest and hence MHWs during this season will have the largest impacts on macroalgal survival. MHW event data were screened, based on their mean intensity as per Hobday et al. (2018), to determine the two most severe events at each location (Table 2), with these two events used to evaluate the potential effects of past MHWs. The intensity of MHWs was the sea surface temperature anomaly (°C) above the long‐term climatological mean for each location (1982–2011), while MHW category was the multiple of the local difference between the climatological mean and the climatological 90th percentile (Hobday et al. 2018).

**TABLE 2:** Marine heatwaves (MHWs) for target macroalgal species.

Species	Equatorward range limit (°S)	MHW start	MHW duration (days)	Mean intensity	Maximum intensity	Cumulative intensity	MHW category
***E. radiata***	**−28.6**	**29/02/2020**	**11**	**1.379**	**1.83**	**15.17**	**I Moderate**
		27/12/2020	20	1.292	1.69	25.84	I Moderate
		**21/02/2024**	**21**	**1.535**	**1.86**	**32.24**	**I Moderate**
		5/01/2025	13	1.162	1.47	15.10	I Moderate
*P. comosa*	−31.6	6/02/2019	14	1.407	1.81	19.70	I Moderate
		**3/03/2020**	**14**	**2.026**	**2.63**	**28.37**	**II Strong**
		2/01/2021	12	1.802	2.12	21.63	I Moderate
		2/03/2021	20	1.74	2.4	34.80	I Moderate
		**24/01/2022**	**20**	**2.12**	**3.39**	**42.40**	**II Strong**
***Durvillaea***	**−36.6**	**15/01/2021**	**20**	**2.624**	**3.34**	**52.49**	**II Strong**
		25/01/2022	13	1.698	2.03	22.08	I Moderate
		16/03/2022	12	1.58	1.93	18.96	I Moderate
		25/01/2023	12	1.656	2.18	19.87	I Moderate
		**9/02/2023**	**28**	**1.843**	**2.67**	**51.60**	**II Strong**
		15/03/2023	26	1.712	2.27	44.51	I Moderate
		7/02/2024	16	1.456	1.91	23.30	I Moderate

*Note:* Data extracted from the Marine Heatwave Tracker portal (www.marineheatwaves.org, accessed 20/01/2025), for summers from 2019 to 2024, at the equatorward range edge for each species in eastern Australia. Bold indicates the two most severe MHWs for each species as applied in subsequent analyses.

For the MHW evaluation, daily satellite‐derived SST data spanning the duration of each MHW event (Table 2), as well as the long‐term climatological mean (1982–2011), were extracted throughout the study domain from the Operational SST and Ice Analysis system (OSTIA) available via the Copernicus Marine Environment Monitoring Service (https://marine.copernicus.eu). Given that we assessed the effects of summer MHWs, the long‐term climatological mean was taken as the average summer SST from 1982 to 2011, to be consistent with the baseline applied by the MHW tracker portal. For MHWs, the average of the daily SST was calculated for each event. The long‐term mean summer and MHW average SST were then applied in the best SDM for each species to estimate the short‐term environmental suitability for each species under these conditions. Changes in short‐term environmental suitability due to MHWs in summer were calculated as the difference between environmental suitability under long‐term mean summer SST conditions and the environmental suitability under MHW conditions.

### 2.8 Range Edge Monitoring

Field surveys were conducted at the equatorward range edge for each study species in eastern Australia, to determine whether range retractions have occurred in recent years in response to MHWs. Survey sites were Brunswick Heads (28.6° S) for *E. radiata* , Bonny Hills (31.6° S) for *P. comosa* and Aragunnu (36.6° S) for *Durvillaea* (Figure 1). Brunswick Heads was selected for monitoring *E. radiata* as this site is the equatorward functional *E. radiata* forest occurring in shallow waters (< 20 m depth) in eastern Australia. It should be noted that *E. radiata* also occurs at Moreton Island (27.0° S), to the north of Brunswick Heads. However, *E. radiata* at Moreton Island occurs in a deep water refugia (> 30 m deep), which protects *E. radiata* from rising sea surface temperatures (SST) (Marzinelli et al. 2015; Davis et al. 2021), and thus this site was largely unsuitable for monitoring the impacts of rising SST or MHWs. Bonny Hills and Aragunnu were selected for monitoring *P. comosa* and *Durvillaea*, respectively, as these sites have the current equatorward stands of these species in eastern Australia.

For *E. radiata* , surveys to monitor changes in *E. radiata* percentage cover were conducted annually at Brunswick Heads, from 2019 to 2024 (except 2022). Surveys consisted of seven randomly positioned 200 m towed video transects on the rocky reef at this site, as per Davis et al. (2020). Percentage cover of *E. radiata* was calculated by analysing habitat type for 25 random points, in 25 photo‐quadrats on each transect, using CPCe software (Kohler and Gill 2006). Statistical tests for differences in cover among years were conducted by permutational analysis of variance (PERMANOVA) in Primer 7 (Anderson et al. 2008; Clarke and Gorley 2015).

For *P. comosa* , surveys to monitor changes in percentage cover were conducted at Bonny Hills, from 2019 to 2024 (except 2020). Surveys consisted of 31–54 randomly positioned photo‐quadrats taken along a fixed 50 m transect on the rocky reef at this site. Percentage cover of *P. comosa* was calculated by analysing habitat type for 25 random points in each photo‐quadrat using CPCe software. Snorkel surveys were used to collect these data as *P. comosa* generally occurs at very shallow depths, on reefs which are not suitable for the application of towed video. Statistical tests for differences in cover among years were conducted by PERMANOVA in Primer 7.

For *Durvillaea*, surveys consisting of intertidal searches of reef platforms were conducted annually at Aragunnu, from 2021 to 2024. Surveys consisted of a visual inspection of reef platforms at Aragunnu, with *Durvillaea* classified using a semi‐quantitative scale from 1 to 3, with a value of 1 indicating few isolated plants (< 10) occurring along a restricted length of the shoreline (< 10 m) and a value of 3 indicating numerous dense plants (> 100) occurring over an extensive length of the shoreline (> 100 m). Intertidal searches were used to assess *Durvillaea* as this species predominantly occurs on wave‐exposed intertidal reef platforms which could not be surveyed using either towed video or snorkel transects. Quantitative data could not be collected for *Durvillaea* due to safety concerns with conducting surveys on the exposed wave‐washed reef platforms where this species occurs (Cheshire and Hallam 1988).

## 3 Results

### 3.1 Variables Influencing Species Distributions in Maxent Models

**FIGURE 3:**
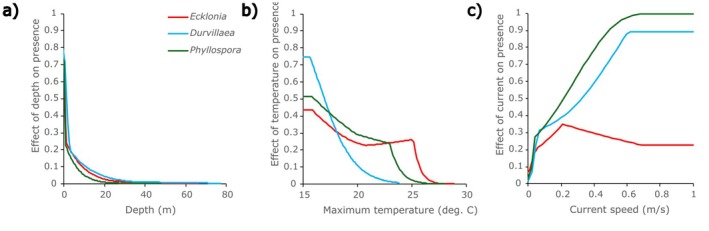
Effects of explanatory variables on macroalgae presence in Australia for the optimal explanatory species distribution models developed. (a) Effect of depth, (b) effect of maximum monthly sea surface temperature and (c) effect of mean annual current speed.

In the Maxent SDMs developed for all species, depth was the most important predictor of macroalgal distributions (Table 3). The relationships between species presence and depth indicated, unsurprisingly, that the predicted probability of macroalgal presence was highest in locations with minimum depths < ~10 m, and rapidly reduced with increasing depth, with very low likelihoods of presence for all species in cells with minimum depths exceeding ~40 m (Figure 3a).

**TABLE 3:** Relative percentage contributions of the environmental predictor variables and thresholds used to define species presence for the best Maxent models developed for macroalgal species in Australia.

Model	Water depth	Long‐term average maximum monthly SST (MaxTemp)	Mean annual current speed (current)
*Ecklonia radiata*	90.5	6.4	3.1
*Phyllospora comosa*	89.0	6.1	4.9
*Durvillaea* spp.	75.0	18.6	6.4

Maximum temperature was also an important predictor in all SDMs (Table 3). For all species, the likelihood of presence showed a non‐linear decline with increasing temperature, with the greatest likelihood of presence occurring in locations with maximum average monthly temperatures of 15°C (the lowest maximum in the study area) and likelihood generally trending downward with increasing maximum average monthly temperature (Figure 3b). The strength of the relationship between species presence and temperature varied among species, as shown by the relative contribution of MaxTemp to each Maxent model, with the effect of maximum temperature being greatest for *Durvillaea* and lowest for *P. comosa* (Table 3). The maximum temperature corresponding to very low likelihood of predicted species presence varied among species, with very low likelihood of *Durvillaea* presence (i.e., < 0.05) predicted for maximum average monthly temperatures above 21.2°C, very low likelihood of *P. comosa* presence above 24.4°C and very low likelihood of *E. radiata* presence above 26.6°C (Figure 3b).

Mean annual current speed also played a role in the prediction of species presence, although the contribution of current to SDMs was lower than that of depth and temperature for all species (Table 3). Generally, there was an increased probability of species presence for cells with higher mean annual current speeds (Figure 3c).

### 3.2 Predictions of Present‐Day (2000–2020) Species Distributions

To ensure overall model validity, and the integrity of the selected presence threshold values, 2000–2020 species distributional data were compared against species presence predictions made by the optimal SDMs for this same period, using both presence threshold values trialled (i.e., ETSS and RL thresholds).

**FIGURE 4:**
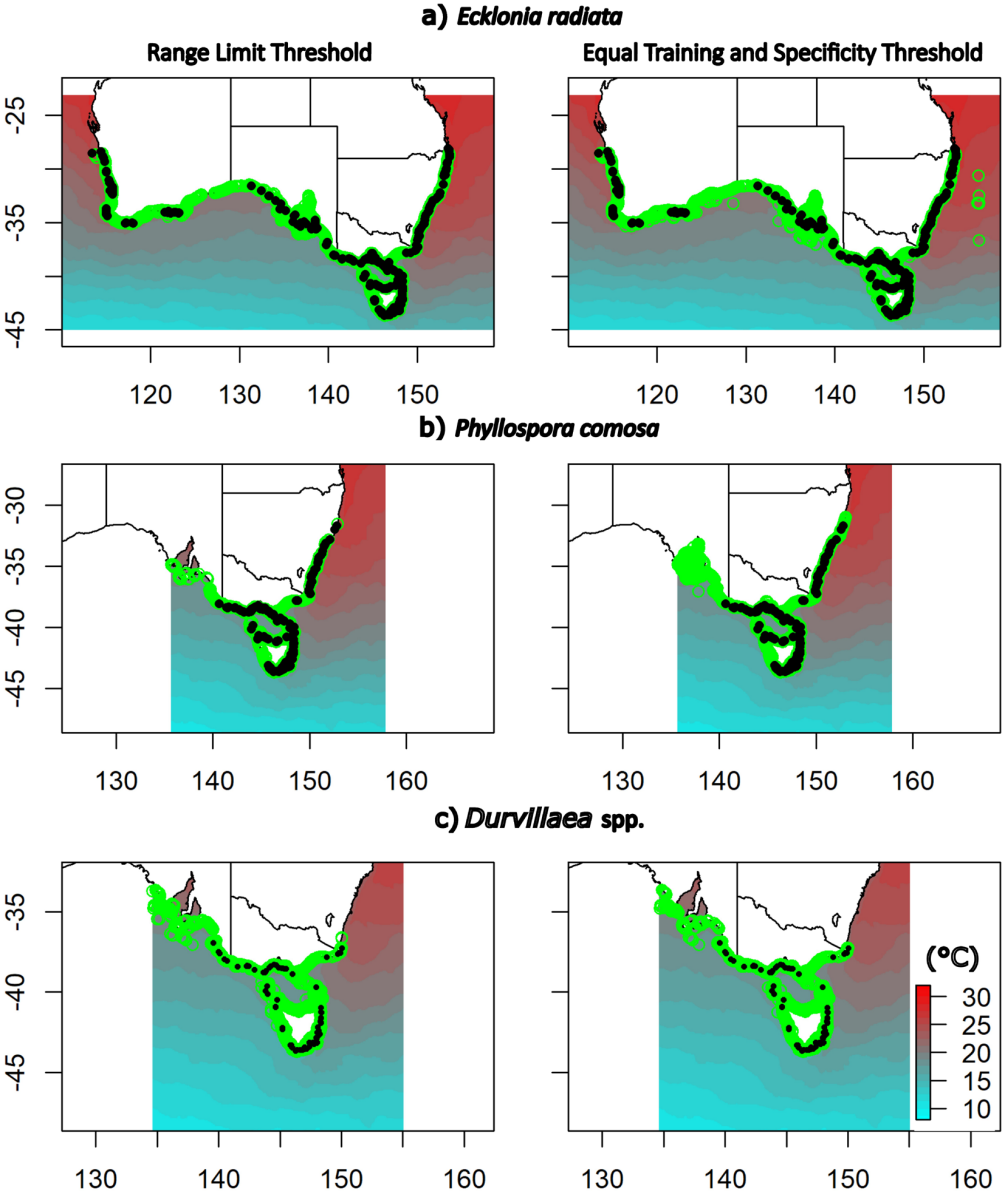
2000–2020 presence records from GBIF (black dots) and predicted distributions of macroalgal species in Australia (green circles) for (a) *Ecklonia radiata*, (b) *Phyllospora comosa* and (c) *Durvillaea* spp. Predictions provided based on Range Limit Threshold (RL, left column) and Equal Training Sensitivity and Specificity Threshold (ETSS, right column). Colour contours show 2000–2020 SST from Bio‐Oracle.

Predicted presences from optimal SDMs for all species applied using both thresholds were closely aligned with realised presence data from GBIF. Species' presence was predicted in a narrow coastal strip along the southern shoreline of Australia and around Tasmania, with the predicted extent of the distribution of each species varying among species, but generally closely matching observed species presence data (Figure 4). For *E. radiata* (Figure 4a) and *P. comosa* (Figure 4b), the RL threshold more accurately modelled distributions, with the ETSS threshold tending to overestimate the extent of the species' distributions at their equatorward range limits (Figure 4a,b). For *Durvillaea* (Figure 4c) both threshold criteria provided similar performances, giving a close match to observed presences at the eastern range edge, while overestimating the extent of species presence at the western range edge.

### 3.3 Predicted and Realised Impacts of Marine Heatwaves

**FIGURE 5:**
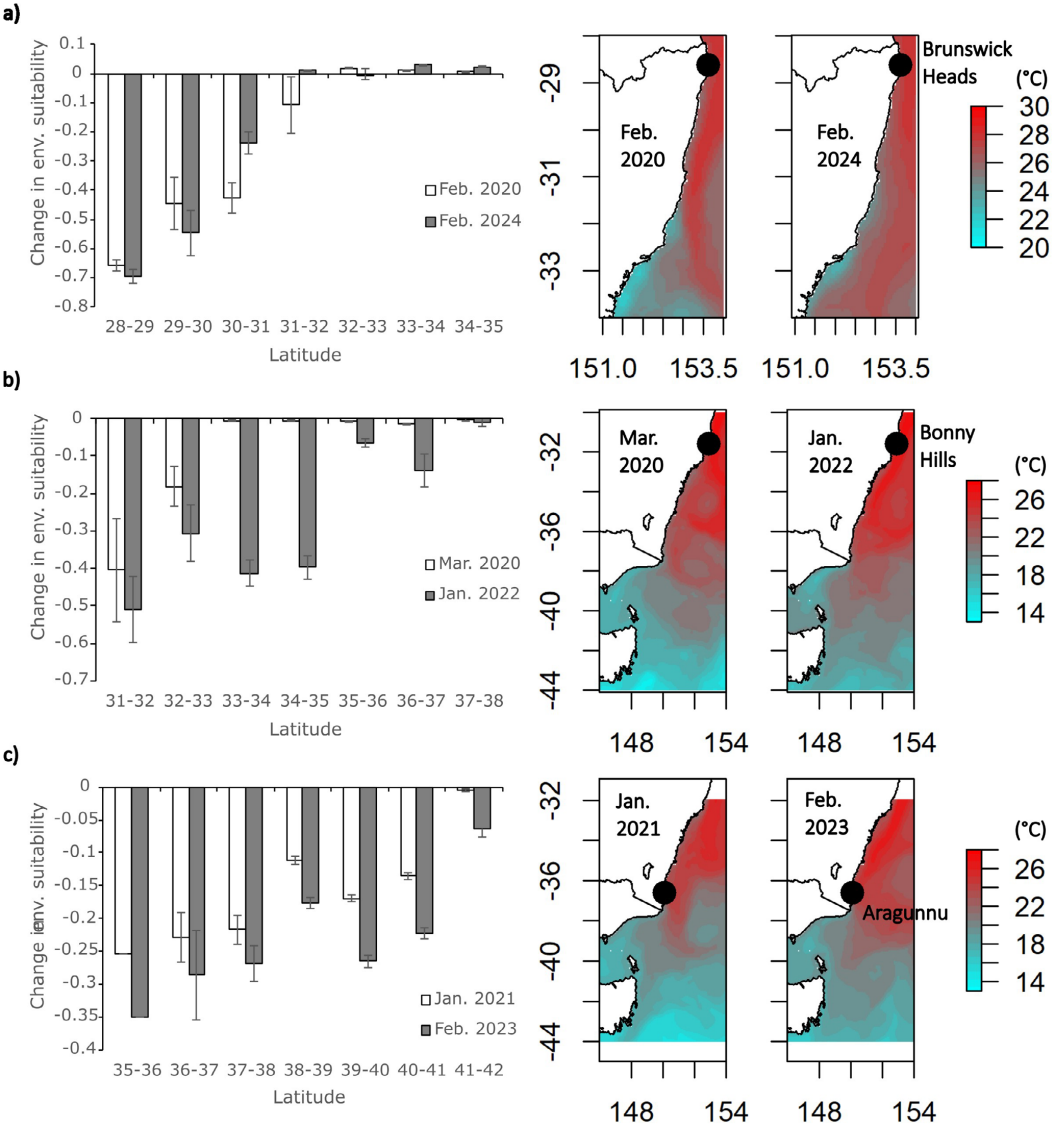
Predicted change in short‐term environmental suitability and associated average SST (°C) during the two most severe marine heatwave events that occurred from 2019 to 2024 at the current equatorward range limit for each species (black circles) for; row (a) *Ecklonia radiata*, row (b) *Phyllospora comosa* and row (c) *Durvillaea* spp.

**FIGURE 6:**
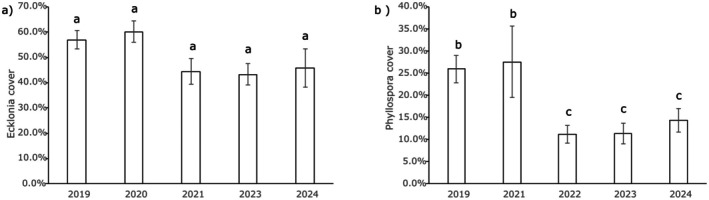
Average habitat % cover on monitored transects for (a) *Ecklonia radiata* at Brunswick Heads and (b) *Phyllospora comosa* at Bonny Hills (mean ± SE). Differing letters (b, c) indicate significant differences among years.

For *E. radiata* , moderate category (intensity 1.0–2.0) MHWs occurred four times at Brunswick Heads from 2019 to 2024, with the two most severe events occurring in February 2020 and February 2024 (Table 2). These moderate MHWs were predicted to reduce the short‐term environmental suitability for *E. radiata* across an area spanning ~3.0° of latitude, with environmental suitability reduced by 50%–70% at the equatorward range edge for the species for both MHWs (Figure 5a). At higher latitudes (30°–32° S), the MHW in February 2020 was predicted to have caused more severe reductions in short‐term environmental suitability, with these more severe reductions predicted to occur due to higher SST adjacent to the coast in 2020 at these latitudes when compared against those for the 2024 MHW (Figure 5a). However, surveys examining the actual impacts of MHWs on *E. radiata* at Brunswick Heads, since 2019, identified no significant variations in *E. radiata* cover among years (*p* = 0.082, Figure 6a), with *E. radiata* still present at this location in 2024, despite increasing SST and four MHWs lasting > 10 days since 2019 (Table 2).

For *P. comosa* , moderate category MHWs occurred three times at Bonny Hills from 2019 to 2024, while two strong category (intensity 2.0–3.0) MHWs occurred in March 2020 and January 2022 (Table 2). The March 2020 MHW was predicted to reduce short‐term environmental suitability for *P. comosa* across an area spanning ~2.0° of latitude, with environmental suitability reduced by 20%–40% at the equatorward range edge for the species (Figure 5b). More widespread effects at higher latitudes were predicted for the more severe MHW that occurred in January 2022. This was predicted to have reduced the short‐term environmental suitability for *P. comosa* across an area spanning ~6.0° of latitude, with environmental suitability reduced by 30%–50% at the equatorward range edge for the species due to high SST extending to higher latitudes in the 2022 event (Figure 5b). Surveys examining the actual impacts of MHWs on *P. comosa* at Bonny Hills since 2019 identified that *P. comosa* was still present at this location in 2024, despite increasing SST and five MHWs since 2019. However, significant variations in *P. comosa* cover were detected among years (*p* < 0.001), with significantly lower cover in 2022–2024 than in 2019–2021 (*p* < 0.039 all comparisons), but no significant differences between cover in 2019 and 2021 (*p* = 0.838) or among cover in 2022, 2023 and 2024 (*p* > 0.351 for all tests, Figure 6b).

For *Durvillaea*, moderate category MHWs occurred five times at Aragunnu from 2019 to 2024, with two strong MHWs occurring in January 2021 and February 2023 (Table 2). These MHWs were of similar magnitude and were predicted to reduce the short‐term environmental suitability for *Durvillaea* across an area spanning ~6.0° of latitude, with environmental suitability reduced by 40%–50% at the equatorward range edge for the species (Figure 5c). The areas where short‐term environmental suitability was reduced for *Durvillaea* under MHW conditions were predicted to be widespread and included the equatorward range edge in NSW extending down to Tasmania, indicating that *Durvillaea* may be sensitive to MHWs across a large geographical area (Figure 5c). Surveys examining the actual impacts of MHWs on *Durvillaea* at Aragunnu, from 2021 to 2024, identified that *Durvillaea* was still present at this location in 2024, despite increasing SST and seven MHWs since 2019, including two MHWs categorised as strong (Table 2). *Durvillaea* maintained a semi‐quantitative score of 3.0 across all years, indicating that there were numerous plants (> 100) occurring over an extensive length of the shoreline (> 100 m) at this site, with no visually apparent change in abundance or cover over time.

### 3.4 Future Projections of Macroalgal Loss

Future projections, made using the best SDM for each species, indicated that all species are expected to undergo areal losses and range contractions under future climate change scenarios. The magnitude of the projected losses and contractions was dependent on the species, the climate change scenario and the threshold used to define species presence (Table 4).

**TABLE 4:** Area of 0.05° cells where species are predicted to be present in Australia under current (2000–2020) and future (2090–2100) conditions, percentage reduction in areal extent for future scenarios and projected range contractions in eastern (E) and western (W) Australia.

Species	Year (scenario)	2000–2020		2090–2100 (RCP2.6)		2090–2100 (RCP6.0)		2090–2100 (RCP8.5)	
		(ETSS)	(RL)	(ETSS)	(RL)	(ETSS)	(RL)	(ETSS)	(RL)
*E. radiata*	Area (km^2^)	60,619	47,497	60,064	47,066	54,089	42,877	50,454	37,948
	Reduction in area (%)			0.9%	0.9%	10.8%	9.7%	16.8%	20.1%
	Range contraction (E, km)			8	152	352	446	696	801
	Range contraction (W, km)			0	0	24	202	352	413
*P. comosa*	Area (km^2^)	22,763	17,711	21,284	13,922	17,649	6868	10,811	3326
	Reduction in area (%)			6.5%	21.4%	22.5%	61.2%	52.5%	81.2%
	Range contraction (E, km)			0	468	568	679	702	679
*Durvillaea*	Area (km^2^)	18,758	20,791	14,138	16,386	3973	6838	0	677
	Reduction in area (%)			24.6%	21.2%	78.8%	67.1%	100.0%	96.7%
	Range contraction (E, km)			147	119	735	713	832	832

*Note:* Values calculated using Equal Test Sensitivity and Specificity Threshold (ETSS) and Range Limit Threshold (RL). Future projections are provided for climate change scenarios RCP 2.6, 6.0 and 8.5.

**FIGURE 7:**
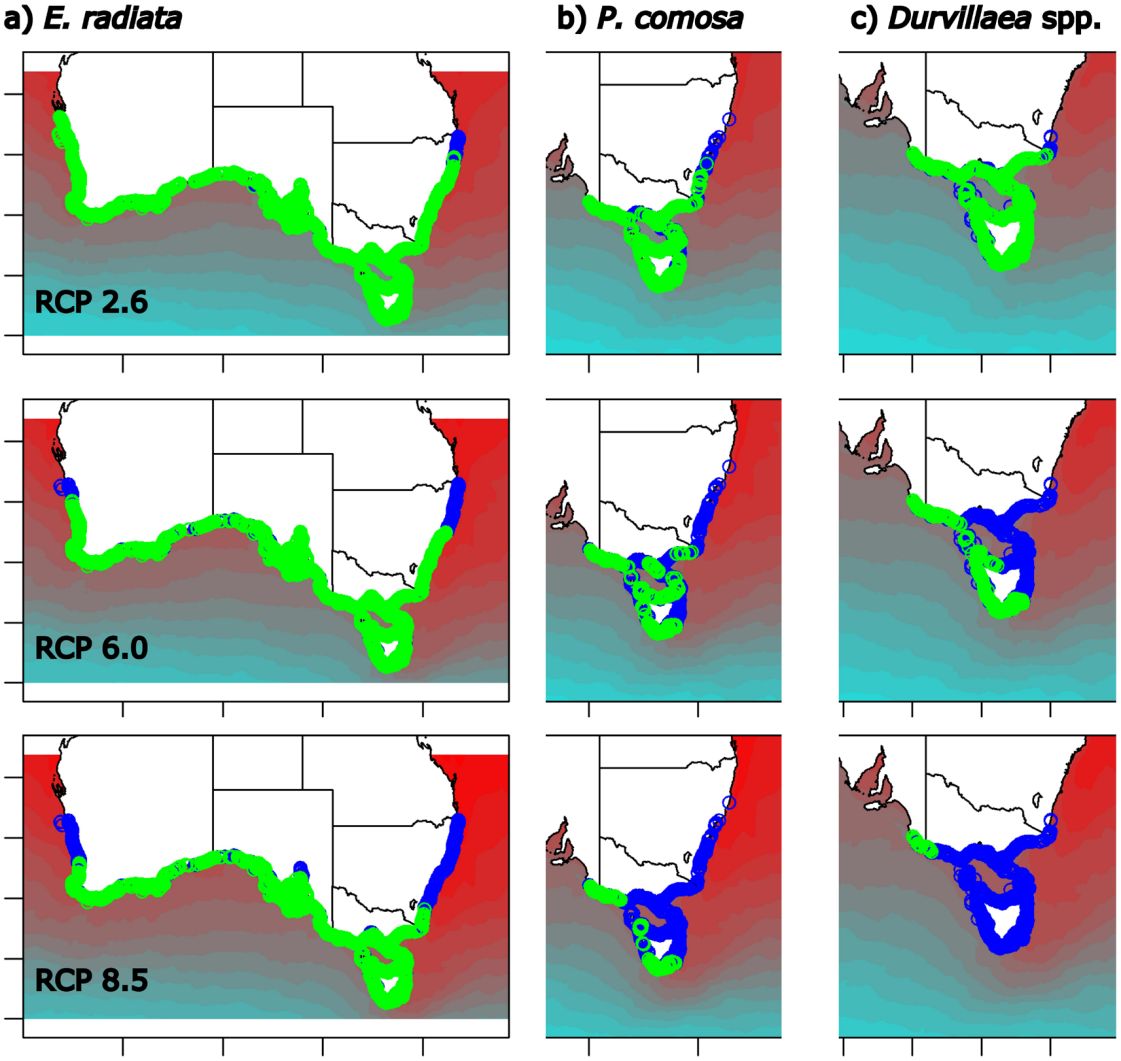
Future projections (2090–2100) for the distribution of (a) *Ecklonia radiata*, (b) *Phyllospora comosa* and (c) *Durvillaea* spp. in Australia based on Range Limit Threshold. Projected future distribution (green circles) and projected habitat loss (blue circles). Colour contours show future projected SST from Bio‐Oracle.

For *E. radiata* , estimates for future reductions in areal extent for the Australian continent ranged from 0.9% (RCP2.6) to 20.1% (RCP 8.5), with losses increasing with the severity of the climate change scenario examined (Table 4). These losses were primarily projected to occur at lower latitudes on both the east and west coasts of Australia (Figure 7a), with losses on the east coast projected to lead to range contractions ranging from 8 km (RCP2.6, ETSS) to 801 km (RCP8.5, RL, Table 4, Figure 7), with slightly lower range contractions projected for the west coast (0–413 km, Table 4). Generally, the RL threshold (0.407) provided more extreme (i.e., greater) projections for habitat losses and range contractions than the ETSS threshold (0.190).

For *P. comosa* , projected reductions in areal extent ranged from 6.5% (RCP2.6) to 81.2% (RCP 8.5, Table 4). Losses increased with the severity of the climate change scenario examined and were primarily projected to occur at lower latitudes in New South Wales (NSW) for RCP2.6, while for RCP 6.0 and 8.5 losses were also projected on the south coast and around Tasmania (Figure 7b). Losses were projected to lead to range contractions ranging from 0 km (RCP2.6, RL) to 702 km (RCP8.5, RL, Table 4), with range contraction projections for *P. comosa* found to be highly sensitive to the threshold used to define species presence. Under RCP8.5, *P. comosa* was projected to become locally extinct in NSW, for both thresholds, with reduced populations persisting in Victoria, South Australia and Tasmania (Figure 7b). Generally, the RL threshold (0.594) provided more extreme (i.e., greater) projections for habitat losses and range contractions than the ETSS threshold (0.214).

For *Durvillaea*, projected reductions in areal extent ranged from 21.2% (RCP2.6, RL) to 100% (RCP 8.5, ETSS, Table 4). Losses increased with the severity of the climate change scenario examined and were projected to occur at both the eastern and western range edges for the species on Australia's south coast for RCP2.6 and also on Tasmania's north and east coasts for RCP6.0 (Figure 7c). For RCP 8.5, *Durvillaea* was projected to become extinct (100% loss) in Australia based on projects with the ETSS threshold, while projections for the RL threshold indicated that *Durvillaea* is projected to become locally extinct in Tasmania and NSW, with only small remnant populations projected to persist on the mainland between Victoria and South Australia (Figure 7c). These losses were calculated to correspond to range contractions on the east coast ranging from 119 km (RCP2.6, RL) to 832 km (RCP8.5, Table 4), which is equivalent to a complete loss of this species along this coastline. In contrast to the other species, the ETSS threshold (0.224) provided more extreme (i.e., greater) projections for habitat losses and range contractions than the RL threshold (0.162).

## 4 Discussion

MHWs and rising ocean temperatures have not caused range contractions for the canopy‐forming macroalgal species, *E. radiata* , *P. comosa* and *Durvillaea* in eastern Australia in recent years (2019–2024) despite modelling suggesting that range contractions could have occurred. This indicates that these species can withstand moderate and strong MHWs at their range edge and suggests that reliance on modelling, without support from field surveys, may lead to overestimation of the potential for future range contractions in response to short‐term thermal extremes. Our projections of future range contractions for these species were substantial, but are lower than those previously reported (e.g., by Martínez et al. 2018), thus demonstrating the value of validating models using extant empirical data to assess the true resilience of species to long‐term warming and short‐term MHWs. Despite the variations in projections of loss among studies, substantial range contractions are still projected for all three species under future climate change scenarios which must be anticipated and managed for.

### 4.1 Observed Impacts of Marine Heatwaves and Ocean Warming

Our monitoring data revealed that *E. radiata* and *P. comosa* have not experienced substantial poleward range retractions over recent years (2019–2024) despite several moderate and strong MHWs occurring during this period. Contrastingly, prior to 2001, *Durvillaea* may have experienced a ~20 km range retraction, from Bermagui (36.4° S) to its current equatorward range limit at Aragunnu (36.6° S). This range retraction occurred sometime prior to 2001 and was attributed to rising ocean temperatures (Millar 2001), with ocean temperatures rising by ~1.5°C off eastern Australia since 1940 (Richardson et al. 2020). Since 2001, *Durvillaea* has experienced no further range retractions, despite repeated moderate–strong MHWs occurring at the equatorward range edge for the species during summer, including the longest (> 35 weeks) and most intense (maximum intensity 2.9°C) MHW ever recorded in the Tasman Sea in 2015/2016 (Oliver et al. 2017). This suggests that potentially only MHWs at the extreme level are likely to precipitate the loss of this genus, and no extreme events have occurred off this section of coast since 1980.

Similarly, we found that *E. radiata* has not experienced range retractions at its equatorward range edge in NSW (Brunswick Heads, 28.6° S), despite repeated moderate (1.47°C–1.86°C) MHWs in summer since 2019. Some localised losses have been reported in equatorward NSW for *E. radiata* , particularly around Coffs Harbour (30° S), with these losses attributed to warming and associated tropical fish herbivory (Vergés et al. 2016), rather than to MHWs which were not assessed. *Phyllospora comosa* has also not undergone range retractions at its equatorward range edge (Bonny Hills, 31.6° S) since 2019, despite repeated moderate–strong MHWs. While localised losses have occurred for *P. comosa* around Sydney (33.8° S), these losses were attributed to historical issues with poor water quality (Coleman et al. 2008) and not MHWs or rising ocean temperatures, although the potential contribution of these effects to losses was not assessed. Consequently, we conclude that there have not been recent range retractions for any of the three main canopy forming macroalgal species in eastern Australia, despite repeated moderate–strong MHWs and ocean warming. This finding suggests that moderate–strong (intensity 1.0°C–3.0°C) MHWs currently have limited impact on the distributions of these major habitat‐forming species. This indicates that these species could have developed strong adaptive capacity to cope with acute thermal events at their range edge, with both *P. comosa* and *E. radiata* showing signatures of selection for warmer temperatures at their range edges (Wood et al. 2021; Vranken et al. 2021; Minne et al. 2025). However, more severe MHWs may still cause losses as was seen off the coast of Western Australia in 2011 (Wernberg et al. 2016; Wernberg 2021), with rising ocean temperatures likely to worsen these impacts. Alternatively, MHWs may be slowly eroding population resilience (e.g., via genetic erosion; Coleman, Minne, et al. 2020; Gurgel et al. 2020) with the visible impacts of MHWs yet to manifest. There is the potential that we missed minor mortality events, and that recovery of cover occurred in between our sampling times (Coleman, Minne, et al. 2020; Coleman, Wood, et al. 2020) or that we missed MHW induced losses at sites south of our range‐edge monitoring locations, although we find this unlikely given there were no observations of such losses. Thus, based on their continued presence following MHWs, the max SST that these species can withstand at their range edges (at least for short periods of ~10 days) is at least 27.4°C ( *E. radiata* ), 25.6°C (*P. comosa*) and 23.1°C (*Durvillaea*) which is 0.8°C, 1.2°C and 1.9°C higher than the species presence data would suggest, respectively.

### 4.2 Potential Future Impacts of Marine Heatwaves

Although moderate–strong MHWs have not substantially impacted habitat‐forming macroalgae at their equatorward range edges in eastern Australia to date, future more severe MHWs with higher intensities (> 3.0°C), which are predicted under climate change (Gregory et al. 2024), may cause more significant negative impacts. MHWs have the potential to cause loss of canopy‐forming macroalgae over large areas, with extreme MHWs linked to substantial declines in macroalgal species globally (Wernberg 2021; Smith et al. 2024). Impacts from MHWs have already manifested for *E. radiata* in Western Australia, where a prolonged extreme MHW over the summer of 2011 (> 10 weeks with maximum intensity 6.7°C) caused the loss of *E. radiata* along > 100 km of coast and had widespread impacts on *E. radiata* health over a much wider area (Wernberg et al. 2016). Our modelling suggests that MHWs temporarily reduce the probability of presence, for all the target macroalgal species examined, over substantial areas in eastern Australia. Consequently, future high‐intensity MHWs (> 3.0°C), particularly those occurring over summer, may negatively impact these species. Understanding where the MHW tipping point for survival lies for these species will be important for anticipating losses and range contractions.

Thus, the magnitude of MHW impacts on macroalgae will depend upon many factors including the intensity and duration of future MHWs (Hobday et al. 2018) and the adaptive capacity of species and populations (Starko et al. 2024). Research indicates that the negative impacts of MHWs on macroalgae, such as bleaching and reduced photosynthetic activity, generally increase with both the intensity and the duration of the elevated temperatures (Straub et al. 2021; Wernberg and Straub 2024). Nevertheless, some macroalgal species can tolerate short periods (1–9 days) of elevated SST without harm (Pereira et al. 2015; Saha et al. 2020; Britton et al. 2020). However, as MHW intensities increase, a thermal tolerance is typically reached, above which bleaching and mortality occur (Pereira et al. 2015; Straub et al. 2021). For example, experimental assessments of the impacts of MHWs on *E. radiata* identified minimal bleaching after 15 days at 25°C, but at 28°C bleaching started within 2–4 days and increased in severity with increased exposure (Wernberg and Straub 2024). Similarly, Straub et al. (2021) examined MHW impacts on *P. comosa*, finding a simulated MHW at 27°C was highly detrimental, causing bleaching followed by mortality within 6–10 days. As far as we could ascertain, no experimental studies of MHW or warming have been done on *Durvillaea*. Another consideration is that for species that occur in the shallow subtidal‐intertidal interface, such as *Durvillaea* and *P. comosa* in NSW, the impacts of MHWs may be compounded by terrestrial heatwaves, with high air temperatures shown to impact the survival of intertidal macroalgal species (Román et al. 2020). Conversely, regular exposure to high air temperatures (relative to lower maximum SSTs) may provide exceptional resilience and adaptive capacity to withstand MHWs. Further experimental research is needed to quantify how different intensities and durations of MHWs will impact macroalgal species at their equatorward range edges in Australia and examining how combined marine and terrestrial heatwaves will impact *P. comosa* and *Durvillaea*. These data are essential to inform managers about the likely impacts of future MHWs, of different intensities and durations, on the critical canopy‐forming macroalgal species that occur within their regions.

### 4.3 Projected Future Impacts of Ocean Warming

The distributions of marine macroalgae are heavily influenced by ocean temperatures (Martínez et al. 2018; Wernberg et al. 2019) with sustained rises in ocean temperatures having the potential to cause substantial changes to species distributions (Takao et al. 2015; Davis et al. 2021). However, no extant range contractions have manifested to date in eastern Australia for *E. radiata* and *P. comosa* , and only a small range contraction (~20 km) has occurred for *Durvillaea* (Millar 2001). Nevertheless, all three of these species are projected to undergo substantial range contractions by 2100 under modelled future climate change scenarios. The magnitude of these projected range contractions varies among species, climate change scenarios, thresholds used in models and studies. Overall, the greatest future range contractions in the current study (832 km) are projected to occur for *Durvillaea*, under the most severe climate change scenario examined (RCP8.5 by 2100), which equates to extinction of this species from most of the Australian continent. The higher sensitivity of *Durvillaea* to ocean warming in models, compared to *E. radiata* and *P. comosa* , is not surprising, given the lower thermal tolerance of this species and that *Durvillaea* is the only one of these species to have exhibited substantial range contractions in eastern Australia to date (Millar 2001). Projections are less dire for the RCP2.6 and RCP6.0 scenarios, with *Durvillaea* projected to remain throughout most of its current range under RCP2.6, while under RCP6.0 it is projected to undergo range contractions of ~740 km on Australia's east coast. Overall, our future projections are substantially more optimistic than those of Martínez et al. (2018), who projected complete loss of *Durvillaea potatorum* (here modelled as *Durvillaea* spp.) in Australia by 2100, under both the RCP2.6 and RCP6.0. The differences in future projections among studies indicate that projections can be sensitive to modelling assumptions. The current study differs from the study by Martínez et al. (2018) in terms of the spatial extent of models, the explanatory variables selected to model species distributions and the time periods over which environmental and species occurrence data were collected. Nevertheless, both studies project substantial future range contractions for *Durvillaea* spp., with any range contractions a cause for concern, given that *Durvillaea* supports a diverse range of fauna (Davis et al. 2024) and has cultural significance (Thurstan et al. 2018).

*Phyllospora comosa* is also projected to undergo severe losses by 2100 under RCP8.5 (~54%–82%), with these losses potentially leading to localised extinction of *P. comosa* throughout NSW, eastern Victoria and along the north and east coast of Tasmania. Losses under RCP2.6 (~6%–24%) and RCP6.0 (~25%–63%) are projected to be lower than for RCP8.5, yet *P. comosa* is still expected to undergo substantial range contractions, of up to ~700 km, on Australia's east coast under these scenarios. These losses are also substantially lower than those projected by Martínez et al. (2018), who projected that *P. comosa* would decline by 87% under RCP2.6 (cf ~6%–24%) and 100% under RCP6.0 (cf ~25%–63%) by 2100. These variations in projections from different models, for the same species, highlight the importance of refining future projections to provide a better understanding of the sensitivity of these projections to modelling assumptions and demonstrate the value of comparing projections against observed changes in species distributions. The results of the current study indicate that projections are sensitive to the threshold used to define species presence, highlighting the need to examine the sensitivity of future projected species redistributions to alternative presence–absence threshold values. We also urge authors to present results for three climate change scenarios, rather than pick the most likely one, to aid comparability among studies.

In comparison with the other species examined, *E. radiata* was projected to be least impacted by future climate change, which was expected for this warm‐tolerant kelp (Wernberg et al. 2019). For *E. radiata* , losses by 2100 of only ~4%–5% are projected under RCP2.6, ~13%–14% under RCP6.0 and ~20%–23% under RCP8.5. These losses are projected to lead to range contractions at lower latitudes on Australia's east coast of between 47 and 801 km, with similar range contractions projected to occur on the west coast. For RCP8.5, the projected range contraction on Australia's east coast, of 502–801 km, encompasses the ~530 km contraction projected by 2100 in eastern Australia by Castro et al. (2020). Contrastingly, our projected range contractions are substantially higher than those of Davis, Champion, and Coleman (2022), who calculated contractions of 275 km (cf 502–801 km) in eastern Australia by 2100 under RCP8.5. This difference may, in part, be due to substantial differences in modelling methods, with (Davis, Champion, and Coleman 2022) taking ecological interactions with long‐spined urchins (*Centrostephanus rodgersii*) and temperature variation with depth into consideration in their model. Consequently, reduced range retractions by *E. radiata* were attributed in part to reduced herbivory from urchins on the southern NSW coast, with urchins also projected to undergo a poleward range shift (Davis, Champion, and Coleman 2022). Moreover, projections showed that *E. radiata* will shift to refugia at greater depths (where temperatures are cooler) at mid latitudes as waters warm (Davis et al. 2021; Davis, Champion, and Coleman 2022). Nevertheless, projected losses in the current study are substantially lower than those of Martínez et al. (2018), who projected losses by 2100 of 49% (cf ~4%–5%) under RCP2.6 and 71% (cf ~13%–14%) under RCP6.0. Again, variation among projections of future climate change impacts, from different studies, highlights the importance of refining future projections to reflect real‐world scenarios, especially when these are needed to make management and restoration decisions (Coleman, Wood, et al. 2020; Eger et al. 2021).

## 5 Conclusion

We reveal that modelled range contractions of the key habitat‐forming macroalgal species *Ecklonia radiata*, *Phyllospora comosa* and *Durvillaea* spp. have not been realised despite both long‐term warming and many moderate–strong MHWs in recent decades. This indicates that these key macroalgal species may have greater resilience and adaptive capacity than would be suggested by models. The exception is a limited (~20 km) range contraction by *Durvillaea* reported to have occurred prior to 2001 (Millar 2001). The apparent greater resilience and adaptive capacity of these key macroalgal species is not usually accounted for in projections of future loss, which are likely overestimated. Refining future projections to encompass realised temperature thresholds as well as consider factors such as ecological interactions and adaptive capacity will be vital for informing future conservation and management actions. The finding that these key taxa can withstand moderate–severe MHWs does, however, allow marine estate managers to tailor and prioritise their responses to these extreme events (Champion and Coleman 2025). For example, MHW response actions for these taxa could only be implemented for strong‐extreme MHWs in NSW, freeing up resources to respond to impacts on other priority species.

Regardless of variation among studies and climate projections used, *E. radiata* , *P. comosa* and *Durvillaea* spp. are all projected to undergo moderate to severe range contractions in Australia under future climate change scenarios due to ocean warming, with these impacts likely to be compounded by the occurrence of more frequent and intense MHWs. Marine managers should proactively prepare for these large changes by having management and conservation strategies that consider these future scenarios (e.g., Coleman, Wood, et al. 2020; Wood et al. 2024).

## Author Contributions

**T. R. Davis:** conceptualization (equal), data curation (equal), formal analysis (equal), investigation (equal), methodology (equal), project administration (equal), resources (equal), software (equal), validation (equal), visualization (equal), writing – original draft (equal), writing – review and editing (equal). **C. Champion:** conceptualization (equal), formal analysis (equal), investigation (equal), methodology (equal), software (equal), validation (equal), visualization (equal), writing – original draft (equal), writing – review and editing (equal). **M. A. Coleman:** conceptualization (equal), funding acquisition (equal), project administration (equal), resources (equal), supervision (equal), writing – review and editing (equal).

## Funding

This work was supported by the New South Wales Marine Estate Management Strategy.

## Conflicts of Interest

The authors declare no conflicts of interest.

## Data Availability

The data that support the findings of this study are available from Figshare at https://figshare.com/s/a1da5732ba2a2054a6de.
